# Gender differences in all-cause, cardiovascular and cancer mortality during long-term follow-up after acute myocardial infarction; a prospective cohort study

**DOI:** 10.1186/s12872-017-0508-3

**Published:** 2017-03-14

**Authors:** Kristin Marie Kvakkestad, Morten Wang Fagerland, Jan Eritsland, Sigrun Halvorsen

**Affiliations:** 1 Department of Cardiology, Oslo University Hospital Ulleval, Postboks 4950 Nydalen, 0424 Oslo, Norway; 2 University of Oslo, Postboks 1072 Blindern, 0316 Oslo, Norway; 3 Oslo Centre for Biostatistics and Epidemiology, Research Support Services, Postboks 1110 Blindern, 0317 Oslo, Norway

**Keywords:** Myocardial infarction, Women, Gender, Cardiovascular mortality, Cancer mortality

## Abstract

**Background:**

Gender differences in short-term mortality in acute myocardial infarction (AMI) have been studied extensively, whereas gender differences in long-term mortality and cause of death largely remain unknown. The aim of this study was to assess the long-term risk of all-cause, cardiovascular and cancer death after AMI in women compared to men.

**Methods:**

Consecutive AMI patients were enrolled in a prospective registry between 2005 and 2011. Date and cause of death were obtained by linkage with the Norwegian Cause of Death Registry, with censoring date 31 December 2012. AMI patients with ST-segment elevation (STEMI, *n* = 5159) and without (NSTEMI, *n* = 4899) were analysed separately.

**Results:**

The 5-years all-cause mortality rates in STEMI were 29% in women vs. 17% in men, and 42% vs. 29% in NSTEMI, respectively. After adjustment for age and other confounders, women with STEMI had similar (HR 1.13 [95% CI: 0.98–1.32]) and women with NSTEMI lower (HR 0.82 [95% CI: 0.73–0.92]) risk of long-term all-cause mortality compared to men. Competing-risks analysis showed no significant gender differences in age-adjusted risk of cardiovascular death nor of cancer death. In both genders, the annual risk of cardiovascular death was low after 1 year, but exceeded annual risk of cancer death throughout follow-up.

**Conclusion:**

During long-term follow-up, women with STEMI had similar and women with NSTEMI lower adjusted risk of all-cause mortality compared to men. Age-adjusted risk of death due to cardiovascular disease was similar in both genders and higher than risk of death due to cancer throughout the follow-up period.

**Electronic supplementary material:**

The online version of this article (doi:10.1186/s12872-017-0508-3) contains supplementary material, which is available to authorized users.

## Background

Several studies have shown a higher risk of short-term mortality after acute myocardial infarction (AMI) in women compared to men [1–4], particularly among younger women with ST-segment elevation myocardial infarction (STEMI) [5–7]. Gender differences in survival are observed also in AMI populations treated with percutaneous coronary intervention (PCI) [8, 9]. In other studies, the adjustment for age, cardiovascular (CV) risk factors and treatment have attenuated or eliminated the excess female risk of all-cause mortality [2, 10–13]. Thus, whether or not the underuse of evidence-based treatment can explain the higher mortality in women is an unsettled question.

Little is known about gender differences in all-cause mortality during long-term follow-up of AMI patients (>1 year). Furthermore, causes of death are sparsely documented. A recent study found a temporal switch from predominantly cardiac to non-cardiac causes of death after PCI over two decades [14]. Another observational study suggested a high risk of cardiac death immediately after STEMI, but the risk of death from non-cardiac causes such as cancer increased later during follow-up [15]. Still, a prolonged risk of CV events after a myocardial infarction (MI) have been documented [16], but whether gender differences in causes of death exist during long-term follow-up after AMI is unknown.

The aim of our study was to assess the risk of all-cause mortality in women compared to men during long-term follow-up after AMI, and to study whether there are gender differences in CV and cancer mortality.

## Methods

### Study population

All consecutive AMI patients admitted to Oslo University Hospital (OUH) Ulleval between 1 September 2005 and 31 December 2011 were included in a local AMI registry. Patients were referred to our tertiary centre, with a 24/7 service for PCI, from the region of South-Eastern Norway. The source population and method of registration has been described previously [17]. In brief, all patients admitted to OUH Ulleval alive or with ongoing cardiopulmonary resuscitation and diagnosed with AMI were included in the study. The diagnosis of AMI was based on current international criteria [18]. Patients were categorised as STEMI or non-STEMI (NSTEMI) based on their index electrocardiogram (ECG).

### Data collection and variables

Predefined variables were registered into a case report form by the responsible physician during hospital admission. Trained study personnel checked the report form for completeness and errors before entering the data into an electronic database. A cross check against the hospital discharge register was performed monthly and missing patients were included if they met the diagnostic criteria for AMI [18]. In a random control sample of 200 registered patients, we found >95% correspondence between registered data and the patient records, except for two variables with estimated 8% erroneous values in the registry that were not included in the regression analyses. The frequency of missing data was <7% for each variable in the registry, except for the ‘smoker or ex-smoker’ variable with 13% missing values.

### Treatment and in-hospital mortality

The decision to perform coronary angiography and PCI was made by the treating physician. Coronary angiograms were registered as normal, with atheromatosis, or with significant stenosis defined as >50% narrowing of the lumen in one main coronary vessel or in multiple vessels (including left main stem stenosis). Relevant in-hospital medications and complications, including all-cause death, were registered.

### Follow-up and cause of death

Date and cause of death was obtained by linkage of the local database with the Norwegian Cause of Death Registry containing vital status throughout 2012. Patients were censored if they were alive at the closing date 31 December 2012. Patients who had emigrated at the time of our analyses were censored at the date of last hospital contact (*n* = 80). In the competing-risks analysis of cause-specific mortality, patients were censored if alive at 31 December 2012 or if dead by other causes than the cause-specific analysis. Follow-up time was calculated from admission until censoring or death.

The Norwegian Cause of Death Registry has a 98% coverage of the Norwegian population [19]. For all deaths, a death certificate (paper form IS-1025B) with a logical sequence from the underlying to immediate cause of death must be completed by a doctor. A code from the International Classification of Disease (ICD) system is allocated to the diagnoses in the death certificate. Subsequently the underlying cause of death is identified by the IRIS computer program with the Automated Classification of Medical Entities (ACME) module, or by assessment of a professional coder. CV death was defined as death with underlying diagnoses corresponding to the ICD-10 codes I00-I99, cancer death included cause of death with code C00-C97, and death of other causes included all other underlying causes of death.

### Ethics

Establishment of the local AMI registry and conduction of the study was approved by the Privacy Protection Officer at OUH. The Norwegian Data Protection Authority and the Ministry of Health and Care services provided concession for data linkage with the Norwegian Cause of Death Registry, with an exemption from the requirement of patient consent. The study was submitted to the Regional Committee for Medical Research Ethics (REK), South-East. However, the need for ethics approval from REK was waived according to national regulations (Health Personnel Act §29b). Data were anonymized before analysis.

### Statistical analyses

This was a single-centre prospective cohort study. Only the patient’s first admission between 1 September 2005 and 31 December 2011 was included in each cohort, so the patient was the unit of our analysis. As previous studies found an interaction between gender and type of AMI in relation to mortality [2], we analysed the STEMI and NSTEMI cohorts separately. The primary outcome was all-cause mortality during follow-up, and secondary outcomes were CV and cancer mortality during follow-up.

Differences between women and men in baseline characteristics and treatment were assessed by the Chi-square test for categorical variables and median regression for continuous variables. Odds ratio (OR) of all-cause in-hospital mortality in women compared to men was calculated by logistic regression. Kaplan-Meier survival plots were computed and gender differences in survival assessed with the log-rank test. The crude, age-adjusted and multivariate adjusted hazard ratio (HR) for all-cause mortality in women versus men were calculated by Cox proportional hazards regression. Candidate covariates for the Cox model were age, pre-hospital resuscitation, pre-hospital thrombolysis (STEMI cohort only), previous MI, previous stroke, previous revascularisation, previous peripheral arterial disease, prior hypertension, smoking, diabetes mellitus, coronary angiography, PCI, ventricular tachycardia (VT)/fibrillation (VF) >48 h, cardiogenic shock, atrioventricular block 2^nd^-3^rd^ degree, atrial fibrillation, heart failure, antibiotic treatment, gastrointestinal bleeding and in-hospital stroke. Smoking was considered an important potential confounder, and with 10% (STEMI) and 15% (NSTEMI) of values missing, we used multiple imputation with 11 predictors assumed to be associated with the smoking variable. We tested for an interaction between continuous age and gender in the multivariate Cox model for all-cause mortality. We also compared the HRs for all-cause mortality in women versus men stratified by age (<70 years vs ≥70 years), with a Wald test. The proportional hazards assumption was assessed by plotting the estimated log-log survival functions for men and women against time.

The cumulative incidence function for the probability of cause-specific mortality was stratified by gender [20]. We created a stacked cumulative incidence plot to show how the total probability of one was allocated between all competing events in women and men, including the possibility of survival during follow-up. The Fine-Gray model [21] was applied to find the underlying sub-distribution hazard ratio (sHR) for competing risks of CV, cancer and other-cause mortality in women versus men during follow-up. The sHRs are not cause-specific hazards, but should be interpreted as a binary increased or decreased probability of cause-specific death when adjusted for covariates [22]. All tests were two-sided and a *p*-value <0.05 was considered statistically significant. Analyses were performed with STATA 13 (Statacorp LP, Texas, USA). The study confines with the STROBE (STrengthening the Reporting of OBservational studies in Epidemiology) checklist for reporting of observational studies [23].

## Results

### Study population

**Fig. 1:**
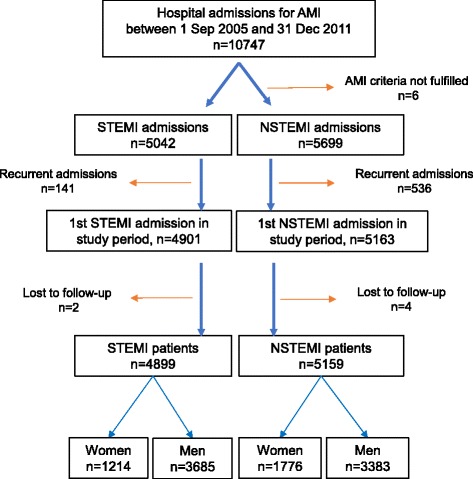
Flow chart. AMI: Acute myocardial infarction

Out of 10 747 registered hospital admissions for AMI, we identified 4899 patients with STEMI (median age 63 years, 25% women) and 5159 patients with NSTEMI (median age 70 years, 34% women) (Fig. 1). Six patients did not fulfill AMI criteria and 677 re-admissions during the period were excluded. Due to typing error in the identification key, six patients could not be linked to the Cause of Death Registry and were lost to follow-up. Baseline characteristics of the population are shown in Table 1.

**Table 1:** Baseline characteristics

	STEMI *N =* 4899			NSTEMI *N =* 5159		
	Women *n* = 1214	Men *n* = 3685	*p*-value	Women *n* = 1776	Men *n* = 3383	*p*-value
Age, years^a^	71 (60–80)	61 (53–70)	<0.0001	77 (65–85)	67 (57–77)	<0.0001
Smoker/ex-smoker, *n* (%)^b^	642 (62.0)	2344 (69.8)	<0.0001	731 (51.0)	1949 (66.1)	<0.0001
Diabetes mellitus, *n* (%)^b^	163 (13.4)	465 (12.6)	0.469	323 (18.2)	659 (19.5)	0.246
Previous hyperlipidemia, *n* (%)^b,c^	148 (12.2)	421 (11.5)	0.468	207 (11.7)	461 (13.7)	0.041
Previous hypertension, *n* (%)^b^	521 (42.9)	1163 (31.6)	<0.0001	892 (50.2)	1320 (39.0)	<0.0001
Previous MI, *n* (%)^b^	145 (11.9)	492 (13.4)	0.198	439 (24.7)	956 (28.3)	0.006
Previous PCI or CABG, *n* (%)^b^	87 (7.2)	428 (11.6)	<0.0001	267 (15.0)	838 (24.8)	<0.0001
Previous stroke, *n* (%)^b^	95 (7.8)	183 (5.0)	0.0002	231 (13.0)	341 (10.1)	0.002
Family history, *n* (%)^b,d^	160 (13.3)	606 (16.4)	0.007	158 (8.9)	438 (13.0)	<0.0001
Peripheral artery disease, *n* (%)^b^	60 (4.9)	143 (3.9)	0.112	121 (6.8)	258 (7.6)	0.276

*MI* Myocardial infarction, *PCI* Percutaneous coronary intervention, *CABG* Coronary artery bypass grafting

^a ^median (25th-75th percentile)

^b ^(%) = percent of patients with available information, denominator may vary

^c ^Hyperlipidemia defined as treatment with lipid-lowering drugs at time of admission

^d ^Coronary artery disease before age 65 years in women, 55 years in men in 1^st^ order relatives

### Treatment and in-hospital mortality

Among STEMI patients, women were less likely to undergo coronary angiography and PCI compared to men, although the percentage receiving angiography was high (>90%) in both genders (Table 2). Women with STEMI were less likely to be treated with antiplatelet therapy, beta-blockers and statins (See Additional file 1: Appendix Table A1). A total of 104/1214 (8.6%) women died in hospital versus 185/3685 (5.0%) of men (OR 1.77 [95% CI: 1.38–2.27]). The gender difference was eliminated after adjustment (age-adjusted OR 1.08 [95% CI: 0.82–1.40]).

**Table 2:** Treatment

	STEMI *N =* 4899			NSTEMI *N =* 5159		
	Women *n* = 1214	Men *n* = 3685	*p*-value	Women *n* = 1776	Men *n* = 3383	*p*-value
Pre-hospital thrombolysis, *n* (%)^a^	105 (8.6)	429 (11.7)	0.004	-	-	-
Coronary angiography, *n* (%)	1115 (91.9)	3591 (97.5)	<0.0001	1200 (67.6)	2925 (86.5)	<0.0001
Normal vessels, *n* (%)^b^	21 (1.9)	52 (1.5)	0.307	157 (13.2)	114 (3.9)	<0.0001
Atheromatosis, *n* (%)^b^	23 (1.9)	48 (1.3)	0.083	126 (10.6)	136 (4.7)	<0.0001
One-vessel disease, *n* (%)^b^	539 (48.4)	1630 (45.5)	0.086	396 (33.2)	1145 (39.4)	0.0002
Multiple-vessel or LMS disease, *n* (%)^b^	530 (47.6)	1853 (51.9)	0.017	512 (43.0)	1513 (52.0)	<0.0001
Missing angiogram, *n* (%)^b^	2 (0.2)	8 (0.2)	NS	8 (0.7)	17 (0.6)	NS
Primary PCI, *n* (%)^c^	808 (66.5)	2653 (72.0)	0.0003	-	-	-
All PCI, *n* (%)	936 (77.0)	3160 (85.7)	<0.0001	524 (29.5)	1598 (47.2)	<0.0001
CABG, *n* (%)	37 (3.0)	187 (5.1)	0.003	109 (6.1)	423 (12.5)	<0.0001
Door to balloon-time, minutes^d^	38 (29–55)	36 (29–52)	0.043	-	-	-
Symptom to balloon-time, minutes^d^	270 (170–495)	245 (155–456)	0.001	-	-	-
Symptom to angiography, days^d^	-	-	-	2 (1–4)	2 (1–4)	<0.0001

*LMS* Left main stem, *PCI* percutaneous coronary intervention, *CABG* Coronary artery bypass grafting

(%) = percent of patients with available information, denominator may vary

^a ^Pre-hospital or in local hospital

^b ^among patients who underwent coronary angiography

^c ^PCI ≤ 12 h from onset of symptoms, without prior thrombolysis

^d ^median (25th–75th percentile)

In the NSTEMI cohort, women were also less likely to undergo coronary angiography and PCI (Table 2) and less likely to receive treatment with antiplatelets and statins compared to men (See Additional file 1: Appendix Table A1). A total of 109/1776 (6.1%) women and 144/3383 (4.3%) of men died in hospital (OR 1.47 [95% CI: 1.14–1.90]). Again, the gender difference was eliminated after adjustment (age-adjusted OR 0.86 [95% CI: 0.65–1.13]). In-hospital complications are shown in Additional file 2: Appendix Table A2.

### Long-term mortality

**Fig. 2:**
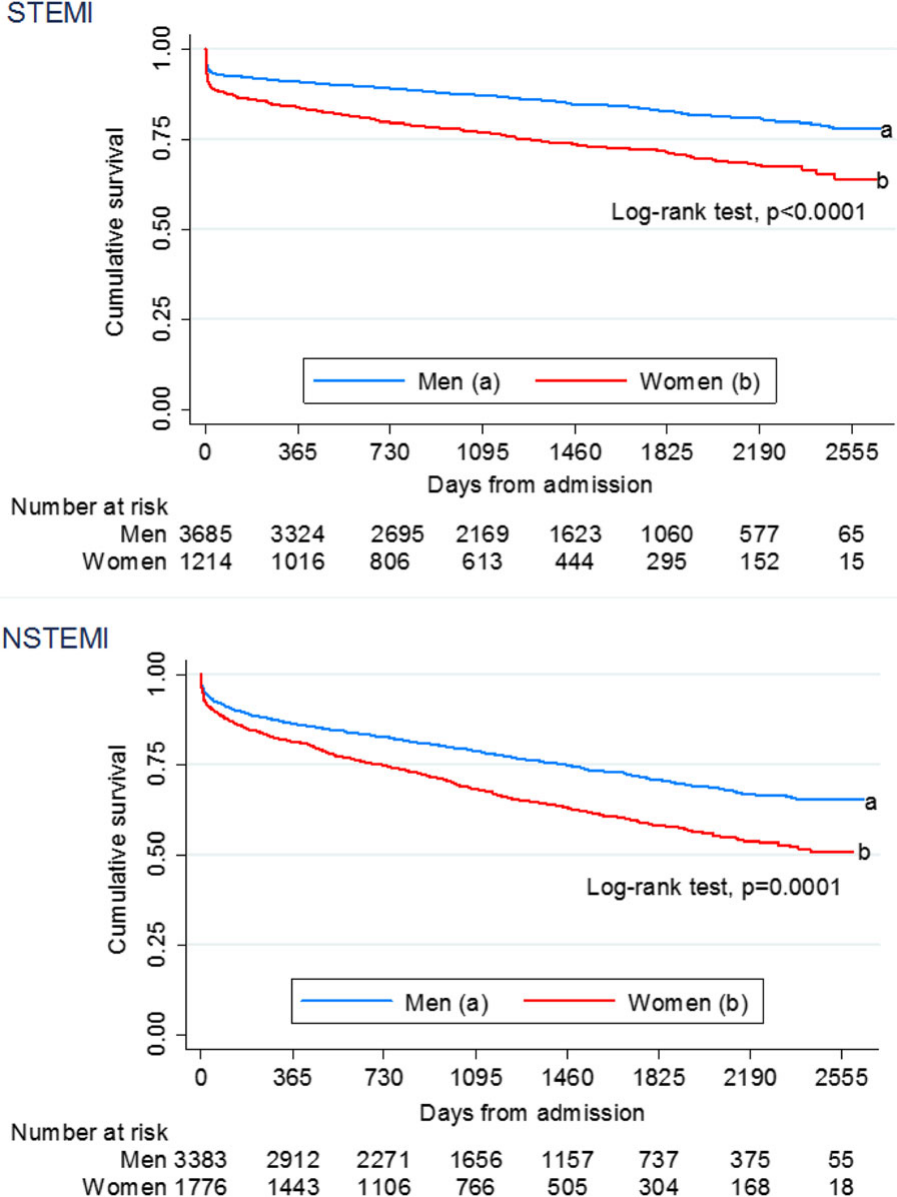
Kaplan-Meier survival estimates of all-cause mortality during follow-up

**Fig. 3:**
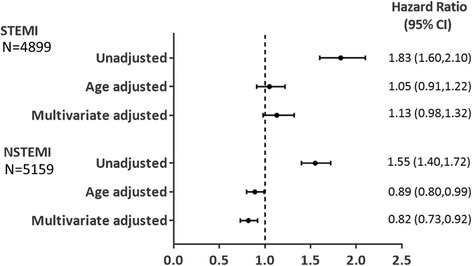
Relative risk of all-cause mortality during follow-up in women compared to men. Median follow-up, STEMI: 1262 days (25^th^–75^th^ percentile: 673–1900), NSTEMI: 1043 days (25^th^–75^th^ percentile: 537–1695). Multivariate adjusted: see Methods section

In the STEMI cohort, 318/1214 (26%) of women and 568/3685 (15%) of men died during a median follow-up of 1262 days (25th-75th percentile [p]: 673–1900). Fig. 2 shows Kaplan-Meier survival plots for women compared to men. The unadjusted HR for all-cause mortality was higher in women compared to men (Fig. 3). After adjustment for age and other confounders, the risk of long-term all-cause mortality was similar in both genders (adjusted HR 1.13 [95% CI: 0.98–1.32]). No gender-age interaction was found (p interaction 0.31).

In the NSTEMI cohort, 631/1776 (36%) of women and 830/3383 (25%) of men died during a median follow-up of 1043 days (25th-75th p: 537–1695, Fig. 2). The unadjusted HR for all-cause mortality was higher in women compared to men, but after adjustment for age and other confounders the risk was lower in women (Fig. 3). No significant interaction was found between continuous age and gender (p interaction 0.052). When stratifying patients into groups <70 and ≥70 years, we found no significant heterogeneity in the association with all-cause mortality (adjusted HR for women versus men <70 years 1.02 [95% CI: 0.77–1.36]; adjusted HR for patients ≥70 years 0.79 [95% CI: 0.70–0.90], *p* = 0.11). The log-log estimated survival curves for men and women were roughly parallel for both STEMI and NSTEMI patients, thus the proportional hazards assumption was regarded valid.

### Cause specific mortality

**Fig. 4:**
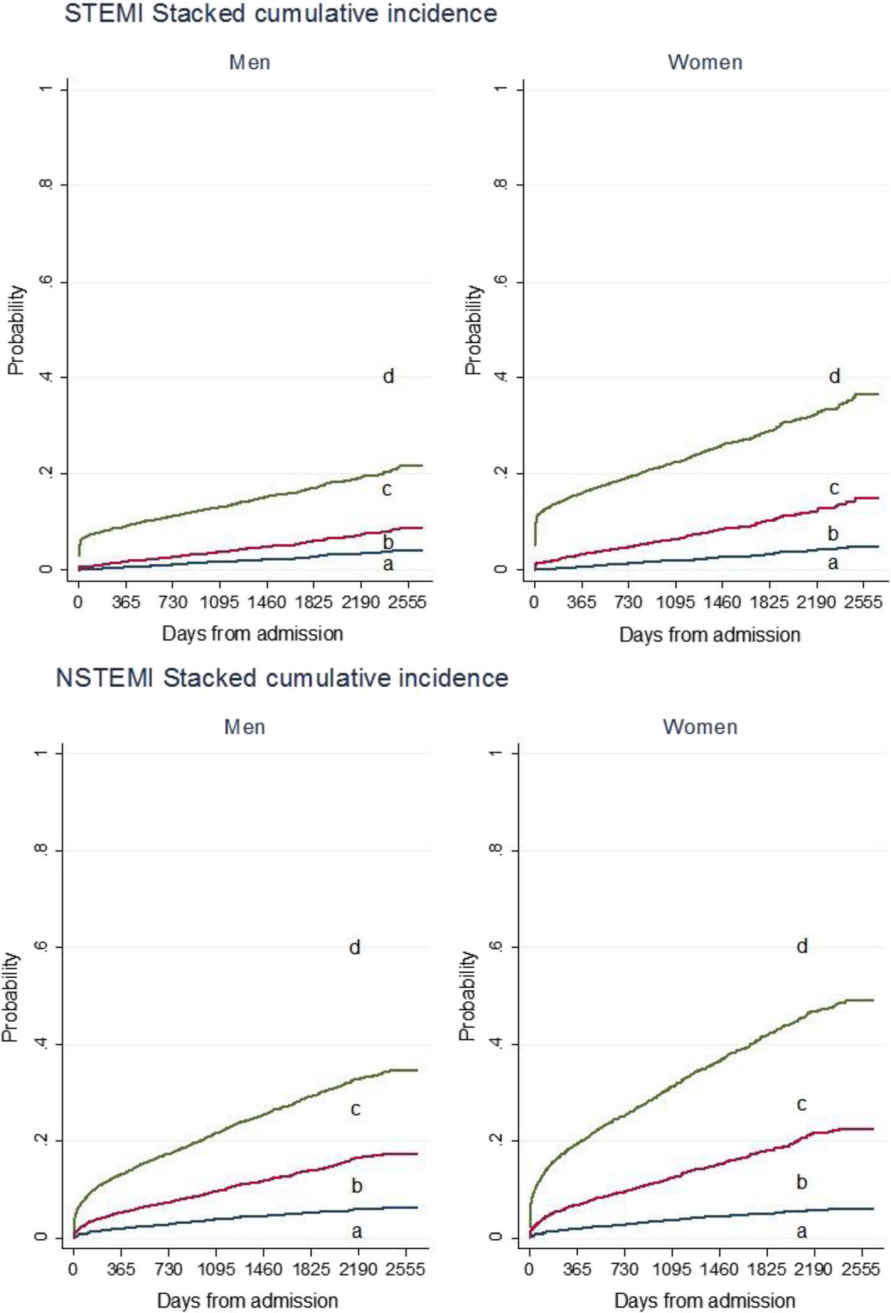
Risk of cause-specific mortality during follow-up in women and men. Stacked cumulative incidence plot. The figure illustrates how the total probability of one was allocated between the competing events: *a*) Cancer death *b*) Other-cause death *c*) Cardiovascular death *d*) Survival during follow-up

Among the 886 STEMI patients who died, CV disease was the cause of death in 67% of patients. Ten percent of women and 15% of men who died, died of cancer. Figure 4 illustrates the stacked cumulative incidence of CV, cancer and other causes of death as a function of time and shows the relationship between the competing causes of death. The calculated sHR confirms the ordering of the cumulative incidence function-plot; women with STEMI were at higher risk of CV mortality than men, but the risk of cancer mortality was similar for both genders during follow-up. After adjustment for age, we found no gender differences in risk of CV nor cancer mortality during follow-up (Table 3).

**Table 3:** Competing risks regression. Sub-distribution hazard ratios of cause-specific mortality during follow-up

	Unadjusted SHR (95% CI)	*p*-value	Age-adjusted SHR (95% CI)	*p*-value
STEMI *n* = 4899				
Cardiovascular death				
Women versus men	1.76 (1.49–2.08)	<0.0001	1.03 (0.87–1.22)	0.750
Cancer death				
Women versus men	1.20 (0.80–1.79)	0.378	0.79 (0.51–1.22)	0.286
Other-cause death				
Women versus men	2.21 (1.63–2.99)	<0.0001	1.26 (0.91–1.74)	0.171
NSTEMI *n* = 5159				
Cardiovascular death				
Women versus men	1.63 (1.42–1.87)	<0.0001	0.97 (0.84–1.12)	0.659
Cancer death				
Women versus men	0.97 (0.74–1.29)	0.852	0.76 (0.56–1.03)	0.072
Other-cause death				
Women versus men	1.52 (1.26–1.84)	<0.0001	0.90 (0.74–1.10)	0.308

*SHR* Sub-distribution hazard ratio, *CI* Confidence interval

Among the 1461 NSTEMI patients who died, CV disease was the cause of death in 58% of women and 54% of men, while cancer was the cause of death in 12% of women and 17% of men. Figure 4 illustrates the relationship between the competing causes of death in NSTEMI patients. After adjustment for age, there were no significant gender differences in risk of CV mortality, but a non-significant reduction of cancer mortality risk in women (sHR 0.76 [95% CI: 0.56–1.03], *p* = 0.072) (Table 3).

In both STEMI and NSTEMI patients the risk of death was highest the first year after AMI (Table 4). After 1 year, the annual CV mortality rate in STEMI was <2.5% in women and <1.5% in men. In NSTEMI patients, the annual CV mortality rate after 1 year was <4.5% in women and <2.5% in men (Table 4). Furthermore, annual CV mortality was higher than annual cancer mortality in both genders throughout the follow-up period (Fig. 4 and Table 4).

**Table 4:** Cumulative mortality during follow-up stratified by gender (Life table method)

	All-cause mortality % (95% CI)		Cardiovascular mortality % (95% CI)		Cancer mortality % (95% CI)	
STEMI^a^	Women	Men	Women	Men	Women	Men
1 year	16.2 (14.3–18.4)	9.2 (8.3–10.1)	12.9 (11.1–15.0)	7.6 (6.8–8.5)	0.7 (0.4–1.4)	0.6 (0.4–0.9)
2 years	20.4 (18.2–22.8)	10.9 (10.0–12.0)	15.1 (13.2–17.3)	8.6 (7.7–9.5)	2.0 (1.3–3.1)	1.1 (0.8–1.5)
3 years	23.0 (20.7–25.6)	13.0 (12.0–14.2)	16.4 (14.4–18.7)	9.6 (8.7–10.6)	2.8 (1.9–4.2)	1.7 (1.3–2.2)
4 years	26.4 (23.8–29.2)	15.3 (14.1–16.6)	18.4 (16.2–20.9)	10.5 (9.5–11.6)	3.6 (2.5–5.1)	2.4 (1.9–3.0)
5 years	28.6 (25.8–31.6)	17.1 (15.8–18.6)	19.5 (17.1–22.2)	11.3 (10.2–12.5)	3.8 (2.7–5.5)	3.0 (2.4–3.8)
NSTEMI^b^	Women	Men	Women	Men	Women	Men
1 year	18.6 (16.9–20.5)	13.6 (12.5–14.8)	12.5 (11.0–14.2)	8.4 (7.5–9.4)	2.0 (1.4–2.8)	2.0 (1.6–2.6)
2 years	25.2 (23.2–27.3)	17.3 (16.0–18.6)	16.2 (14.5–18.0)	10.6 (9.6–11.7)	3.5 (2.7–4.6)	2.8 (2.3–3.5)
3 years	31.9 (29.6–34.2)	21.3 (19.9–22.8)	20.4 (18.5–22.6)	12.4 (11.2–13.6)	4.7 (3.7–6.0)	4.0 (3.3–4.9)
4 years	36.7 (34.3–39.3)	25.2 (23.6–26.8)	23.3 (21.1–25.6)	14.3 (13.0–15.7)	5.5 (4.3–7.0)	5.0 (4.2–6.0)
5 years	42.0 (39.2–44.9)	29.3 (27.5–31.2)	26.2 (23.7–29.0)	16.4 (14.9–18.0)	5.7 (4.5–7.3)	6.3 (5.2–7.5)

*CI* Confidence interval

^a^Women *n* = 1214. Men *n* = 3685. Median (25th-75th percentile) follow-up: 1262 (673–1900) days

^b^Women *n* = 1776. Men *n* = 3383. Median (25th-75th percentile) follow-up: 1043 (537–1695) days

## Discussion

In this long-term follow-up of a large AMI cohort, the main findings were: 1) Women with STEMI had similar and women with NSTEMI better long-term survival compared to men, when controlling for age and other confounding factors. 2) There were no significant gender differences in age-adjusted risk of CV nor cancer mortality.

Long-term follow-up studies after AMI (>1 year) are scarce, but needed. During seven years of follow-up, we show a similar age-adjusted risk of death among women and men with STEMI. A high proportion of both genders were treated invasively, probably contributing to the similar long-term prognosis. These results are in accordance with a recent report from Italy showing similar 1-year mortality for women and men with STEMI [12]. However, other studies of STEMI patients selected for PCI have reported a worse 1-year prognosis in women compared to men, even after adjustment for age and other confounders [8, 9].

Women with NSTEMI had 18% lower risk of long-term death compared to men, after multivariate adjustment. Lower risk in women was found already after age-adjustment, suggesting that age is the most important confounding factor when comparing long-term survival in NSTEMI women versus men. Our results correspond well with data from Sweden, confirming a 1-year survival benefit in NSTEMI women treated during 1998–2002 [24], but contradict several previous studies reporting no gender differences in risk estimates of mortality among NSTEMI patients [12, 13, 25, 26]. These studies differ with regard to study population, study period and confounding factors considered, and a comparison is not straightforward. In general, our study reflects contemporary treatment with a frequent use of invasive treatment also in women, and a much longer follow-up than most other studies. Explanations for better long-term survival in NSTEMI women could be awareness of gender differences in presentation and treatment of AMI resulting in equal opportunities for women and men [27], less extensive coronary artery disease [28] or lower general risk burden compared to their male counterparts [29]. Among NSTEMI women in our study, normal vessels or non-significant coronary artery disease were more prevalent than in men and gender differences in treatment during hospitalization were present. Gender differences potentially exist in complete versus non-complete revascularization, drug prescription patterns, such as duration of dual antiplatelet therapy, drug compliance, and clinical follow-up influencing prognosis. Information about these factors were not available in our study, but should be included in future studies of long-term outcomes in women and men after AMI.

To our knowledge, this is the first study to elucidate the relationship between gender and risk of cause-specific mortality in an unselected AMI population. Especially in ageing clinical cohorts, an increased risk of non-CV death could influence the long-term prognosis after AMI. We did not find any significant gender differences in long-term CV mortality after adjustment for age. After 1 year, the annual CV death rate in STEMI patients in our study was low (<1.5% in men and <2.5% in women), but still higher than the annual cancer death rate throughout the follow-up period. Our results correspond to the results from a Danish study of PCI-treated STEMI patients (mean age 63 years), finding that cardiac risk beyond 30 days post-STEMI was low (<1.5% per year) [15]. In a recent report from Sweden, AMI patients (median age 74 years) who survived 1 year had a 20% risk of non-fatal MI, stroke or CV death during the next 36 months [16]. These studies along with our results all confirm a long-term risk of CV mortality and a continued need for focusing on secondary prevention measures among AMI patients of both genders.

Our finding of a non-significant tendency towards lower age-adjusted cancer mortality in NSTEMI women compared to men needs further investigation. In Norway and Europe, men experience a higher risk of death from cancer compared to women [30, 31], and our results could reflect a higher incidence of and mortality from cancer in men diagnosed with AMI. The results of the present study must be interpreted with caution due to few cancer deaths and the relatively short follow-up period in the setting of a non-cancer cohort. Further studies are needed to register the prevalence and type of cancer in AMI patients, and should be powered to evaluate the risk of cancer death competing with cardiac death during long-term follow-up.

The strengths of our study is a complete follow-up of unselected AMI patients during a 7-year period. We investigated three categories of cause of death, giving a more detailed description of mortality after AMI. We provide a descriptive analysis that indicate contemporary equal opportunities for women and men treated with AMI. Limitations were that several comorbid conditions, such as cancer, renal disease, dementia, autoimmune- and pulmonary diseases were not registered at inclusion, and may have confounded mortality risk. Heart rate and blood pressure was not registered. Follow-up did not include quality of life or physical performance, which are important measures of outcome after AMI. The causes of death in this study were not determined by autopsy, the gold standard for identifying the cause of death. Finally, this was a single centre study from the largest university hospital in Norway, being a referral hospital for 1.5 million people in Eastern Norway. However, the results are not necessarily generalizable to other populations.

## Conclusion

After adjustment for age and other confounding factors, women with STEMI had similar and women with NSTEMI had better long-term prognosis compared to men. There were no significant gender differences in risk of CV nor cancer death during follow-up. After one year, annual risk of CV death in both genders was low, but still exceeded annual risk of death due to cancer. Possible gender differences in long-term risk of cancer death in AMI patients need further investigation.

## Additional files

Additional file 1: Appendix Table A1. Medications in-hospital for women and men with ST-elevation myocardial infarction (STEMI) and non-STEMI (NSTEMI). (DOCX 20 kb)
Additional file 2: Appendix Table A2. Complications in-hospital for women and men with ST-elevation myocardial infarction (STEMI) and non-STEMI (NSTEMI). (DOCX 21 kb)

## Abbreviations

ACME: Automated Classification of Medical Entities
AMI: Acute myocardial infarction
CABG: Coronary artery bypass grafting
CI: Confidence interval
CV: Cardiovascular
ECG: Electrocardiogram
HR: Hazard ratio
ICD: International Classification of Disease
MI: Myocardial infarction
NSTEMI: Non ST-segment elevation myocardial infarction
OR: Odds ratio
OUH: Oslo University Hospital
PCI: Percutaneous coronary intervention
sHR: Sub-distribution hazard ratio
STEMI: ST-segment elevation myocardial infarction
VF: Ventricular fibrillation
VT: Ventricular tachycardia
