# Supplementary material for: Gender differences in all-cause, cardiovascular and cancer mortality during long-term follow-up after acute myocardial infarction; a prospective cohort study
Source: BMC Cardiovasc Disord. 2017 Mar 14;17:75. doi: 10.1186/s12872-017-0508-3 (PMC5348805; doi:10.1186/s12872-017-0508-3)
Supplement: Additional file 1: Appendix Table A1. — Medications in-hospital for women and men with ST-elevation myocardial infarction (STEMI) and non-STEMI (NSTEMI). (DOCX 20 kb) [file 12872_2017_508_MOESM1_ESM.docx]

**Appendix Table A1. Medications in-hospital for women and men with ST-elevation myocardial infarction (STEMI) and Non-STEMI (NSTEMI)**

|  | STEMI N=4899 | | | NSTEMI N=5159 | | |
| --- | --- | --- | --- | --- | --- | --- |
|  | **Women**  **n=1214** | **Men**  **n=3685** | **p-value** | **Women n=1776** | **Men**  **n=3383** | **p-value** |
| Acetylsalicylic acid, n (%) | 1151 (95.4) | 3560 (96.8) | 0.020 | 1535 (87.8) | 3085 (92.8) | <0.0001 |
| ADP-receptor antagonist, n (%) | 1086 (90) | 3452 (93.9) | <0.0001 | 1172 (67) | 2717 (81.7) | <0.0001 |
| Dual antiplatelet therapy, n (%) | 1070 (88.1) | 3436 (93.2) | <0.0001 | 1142 (64.3) | 2674 (79.0) | <0.0001 |
| GP 2b/3a inhibitor, n (%) | 252 (20.9) | 1120 (30.4) | <0.0001 | 49 (2.8) | 212 (6.4) | <0.0001 |
| β-blocker, n (%) | 837 (69.3) | 2806 (76.3) | <0.0001 | 1359 (77.7) | 2663 (80.1) | 0.050 |
| Statins, n (%) | 1003 (83.2) | 3275 (89.0) | <0.0001 | 1234 (70.6) | 2820 (84.8) | <0.0001 |
| Anticoagulation, n (%) | 1013 (83.9) | 3238 (88.0) | 0.0002 | 1159 (66.1) | 2520 (75.2) | <0.0001 |
| Heparin, n (%) | 999 (82.7) | 3189 (86.7) | 0.0005 | 1054 (60.3) | 2386 (71.4) | <0.0001 |
| Vitamin K-antagonist, n (%) | 40 (3.3) | 99 (2.7) | 0.261 | 159 (9.1) | 201 (6.0) | <0.0001 |
| ACE-inhibitor/A2-blocker, n (%) | 412 (34.1) | 1159 (31.5) | 0.091 | 785 (44.9) | 1407 (42.3) | 0.077 |
| Diuretics, n (%) | 363 (29.9) | 703 (19.1) | <0.0001 | 769 (44) | 990 (29.8) | <0.0001 |
| Inotropic agents, n (%) | 138 (11.4) | 297 (8.1) | 0.0004 | 96 (5.5) | 124 (3.7) | 0.003 |
| Antiarrythmic drugs, n (%) | 100 (8.3) | 305 (8.3) | 0.995 | 59 (3.4) | 111 (3.3) | 0.946 |
| ADP: Adenosin diphosphate; GP: Glycoprotein; ACE: Angiotensin converting enzyme; A2: Angiotensin II-receptor  (%) = percent of patients with available information, denominator may vary | | | | | | |
