# Supplementary material for: Gender differences in all-cause, cardiovascular and cancer mortality during long-term follow-up after acute myocardial infarction; a prospective cohort study
Source: BMC Cardiovasc Disord. 2017 Mar 14;17:75. doi: 10.1186/s12872-017-0508-3 (PMC5348805; doi:10.1186/s12872-017-0508-3)
Supplement: Additional file 2: Appendix Table A2. — Complications in-hospital for women and men with ST-elevation myocardial infarction (STEMI) and non-STEMI (NSTEMI). (DOCX 21 kb) [file 12872_2017_508_MOESM2_ESM.docx]

|  | STEMI N=4899 | | | NSTEMI N=5159 | | |
| --- | --- | --- | --- | --- | --- | --- |
|  | **Women n=1214** | **Men**  **n=3685** | **p-value** | **Women n=1776** | **Men**  **n=3383** | **p-value** |
| VT/VF≥48 h | 11 (0.9) | 34 (0.9) | 0.961 | 9 (0.5) | 36 (1.1) | 0.041 |
| Cardiogenic shock | 60 (4.9) | 176 (4.8) | 0.813 | 21 (1.2) | 46 (1.4) | 0.592 |
| AV-block II-III | 69 (5.7) | 118 (3.2) | <0.0001 | 18 (1.0) | 28 (0.8) | 0.501 |
| Atrial fibrillation | 124 (10.2) | 258 (7.0) | 0.0003 | 133 (7.5) | 153 (4.5) | <0.0001 |
| Heart failure | 192 (15.8) | 365 (9.9) | <0.0001 | 305 (17.2) | 281 (8.3) | <0.0001 |
| Cerebrovascular stroke | 19 (1.6) | 19 (0.5) | 0.0003 | 43 (2.4) | 32 (0.9) | <0.0001 |
| GI-bleeding | 20 (1.6) | 41 (1.1) | 0.146 | 22 (1.2) | 31 (0.9) | 0.276 |
| Antibiotic treatment | 151 (12.4) | 304 (8.2) | <0.0001 | 336 (18.9) | 382 (11.3) | <0.0001 |
| In-hospital mortality | 104 (8.6) | 185 (5.0) | <0.0001 | 109 (6.1) | 144 (4.3) | 0.003 |
| VT: Ventricular tachycardia; VF: Ventricular fibrillation; AV: Atrioventricular; GI: Gastrointestinal. All numbers are frequencies (%). | | | | | | |

**Appendix Table A2. Complications in-hospital for women and men with ST-elevation myocardial infarction (STEMI) and Non-STEMI (NSTEMI)**
